# Bridging Genetic Insights with Neuroimaging in Autism Spectrum Disorder—A Systematic Review

**DOI:** 10.3390/ijms25094938

**Published:** 2024-04-30

**Authors:** Joana Vilela, Célia Rasga, João Xavier Santos, Hugo Martiniano, Ana Rita Marques, Guiomar Oliveira, Astrid Moura Vicente

**Affiliations:** 1 Departamento de Promoção da Saúde e Doenças Não Transmissíveis, Instituto Nacional de Saúde Doutor Ricardo Jorge, Avenida Padre Cruz, 1649-016 Lisboa, Portugal; joana.vilela@insa.min-saude.pt (J.V.); celia.rasga@insa.min-saude.pt (C.R.); joao.xavier@insa.min-saude.pt (J.X.S.); hugo.martiniano@insa.min-saude.pt (H.M.); a.rita.marques@insa.min-saude.pt (A.R.M.); 2 BioISI-Biosystems & Integrative Sciences Institute, Faculty of Sciences, University of Lisboa, Campo Grande, C8, 1749-016 Lisboa, Portugal; 3 Unidade de Neurodesenvolvimento e Autismo, Serviço do Centro de Desenvolvimento da Criança, Centro de Investigação e Formação Clínica, Hospital Pediátrico, Centro Hospitalar e Universitário de Coimbra (CHUC), 3000-602 Coimbra, Portugal; guiomar@chuc.min-saude.pt; 4 Coimbra Institute for Biomedical Imaging and Translational Research, University Clinic of Pediatrics, Faculty of Medicine, University of Coimbra, 3000-602 Coimbra, Portugal

**Keywords:** autism spectrum disorder, neuroimaging, neurogenetics, brain imaging, electroencephalography, magnetic resonance imaging, functional magnetic resonance imaging, systematic review

## Abstract

Autism Spectrum Disorder (ASD) is an early onset neurodevelopmental disorder characterized by impaired social interaction and communication, and repetitive patterns of behavior. Family studies show that ASD is highly heritable, and hundreds of genes have previously been implicated in the disorder; however, the etiology is still not fully clear. Brain imaging and electroencephalography (EEG) are key techniques that study alterations in brain structure and function. Combined with genetic analysis, these techniques have the potential to help in the clarification of the neurobiological mechanisms contributing to ASD and help in defining novel therapeutic targets. To further understand what is known today regarding the impact of genetic variants in the brain alterations observed in individuals with ASD, a systematic review was carried out using Pubmed and EBSCO databases and following the Preferred Reporting Items for Systematic Reviews and Meta-Analyses (PRISMA) guidelines. This review shows that specific genetic variants and altered patterns of gene expression in individuals with ASD may have an effect on brain circuits associated with face processing and social cognition, and contribute to excitation–inhibition imbalances and to anomalies in brain volumes.

## 1. Introduction

Autism Spectrum Disorder (ASD) is an early onset neurodevelopmental disorder characterized by impaired social interaction and communication, and restricted and repetitive patterns of behavior and interests [1]. Currently, key areas of research include understanding the complex genetic architecture of ASD, examining the brain networks implicated in its development, assessing patterns of brain connectivity and identifying potential biological markers for early diagnosis and prognosis. However, these lines of research are not frequently integrated, hampering an overarching perspective of the causes and processes underlying ASD.

Although the etiology of ASD is still unclear, large family studies estimate heritability values of approximately 80% for the disorder, providing evidence for a strong role of genetics in its etiology [2,3,4,5]. Individuals with ASD have a broad range of clinical phenotypes and frequently have comorbidities like epilepsy, intellectual disability, language impairments, Attention-Deficit/Hyperactivity Disorder (ADHD) and anxiety. There is strong evidence that genetic factors play a major role in ASD, and genetic studies over the past two decades have been key to the elucidation of the disease etiology. It is known that the genetic architecture underlying ASD involves the interplay of common and rare variation, and research has been focusing on understanding the impact of genetic variation on protein structure and function [6,7,8,9,10]. Hundreds of genes have been associated with ASD, and the list is continuously growing [6,7,8,9,10]. Strong genetic-based evidence points to the involvement of synaptic pathways, neurotransmission, transcription regulation and chromatin modification. Synaptic and neurotransmitter signaling plays a key role in the development of both the peripheral and the central nervous system, and there are multiple lines of evidence from genetic, imaging and functional studies implicating the dysfunction of these mechanisms in ASD pathophysiology. For instance, abnormalities in excitatory neurotransmission involving glutamate receptors genes and the dysregulation of glutamatergic pathways have been reported in individuals with ASD and in animal models for ASD [11]. The observed high prevalence of epilepsy in individuals with ASD, compared to the general population, strengthens the hypothesis of an excitatory and/or inhibitory imbalance contributing to altered brain activity [11,12,13,14,15].

Brain imaging and electroencephalography (EEG) are particularly attractive methodological tools to study alterations in brain structure and function. Neuroimaging techniques have been widely used to study the neuroanatomy and the structural connectivity of the brain in ASD, providing a more accurate assessment of the neuroanatomical underpinnings of the disorder. In combination with neuropathological and clinical research, neuroimaging studies have been very important to identify and characterize the development trajectories in ASD [16,17,18]. Studies using magnetic resonance imaging (MRI) have focused on neuroanatomical features to identify and characterize morphological anomalies within the brain regions of ASD subjects, analyzing cortical and subcortical brain regions, total brain volumes, and volumes of specific brain structures [16,17]. MRI studies in individuals with ASD have indicated atypical frontal and temporal lobe development, reduced gray and white matter volumes, and increased total cerebellar volumes, as well as decreased amygdala volumes in comparison to individuals without ASD. MRI analysis has also been used to study children with early accelerated brain growth. Macrocephaly, which is characterized by a head circumference equal or greater than two standard deviations above average for the age, is one of the most replicated findings in ASD. This brain enlargement has been associated with genetic variants implicating the PI3K/AKT/mTOR pathway, an intracellular signaling pathway with fundamental roles in cell cycle regulation, proliferation, and quiescence [16]. Structural connectivity is being measured using MRI-based techniques that analyze the structural integrity of white matter tracts that constitute structural correlates of brain connectivity [17,18,19,20]. Functional magnetic resonance imaging (fMRI) is used to assess the neural activity and the connectivity among different brain regions. fMRI brain studies have been key for establishing ASD as a disorder involving abnormal or diminished functional connectivity [21] and allowing the establishment of correlations between neurocognitive alterations and anatomical/functional connections. Additionally, a growing body of evidence indicates that individuals with ASD exhibit distinct patterns of brain connectivity when compared to typically developing (TD) individuals [21,22,23,24,25]. All this evidence highlighted the occurrence of altered connectivity, especially between frontal-posterior regions [21,22,23,24,25]. EEG has also been used to examine functional connectivity across different brain regions in ASD, as well as the integrity of structural connections between brain areas [24,26,27].

Integrating brain imaging and EEG studies with genetic studies offers the opportunity of novel insights into the neurobiological processes underlying altered neuroanatomy and brain function in ASD, and how they relate with behavior changes and clinical features that characterize this disorder. This clarification has the potential to define new targets for therapeutic interventions. The current progress in this field was summarized in this systematic review, focusing on findings from studies that combine genetic variation and altered patterns of gene expression with neuroimaging and EEG analysis, and the potential of this approach for improving the understanding of the neurodevelopmental mechanisms underlying ASD was discussed.

## 2. Materials and Methods

A systematic review of the literature was performed following the procedures described in the methodological framework, using the Preferred Reporting Items for Systematic Reviews and Meta-Analyses (PRISMA) standard checklists for reporting results [28,29]. The review aimed at identifying studies that address the connection between genetic and brain alterations, detectable through EEG and magnetic resonance studies (MRI, MRS and fMRS). For this purpose, the following strategy was used: (i) identify the research question, (ii) identify relevant studies, (iii) select studies using inclusion/exclusion criteria, (iv) chart the data, and (v) synthesize findings of the selected studies (results).

### 2.1. Identification of the Research Question

The research question underlying this systematic review was: “Which genetic and brain alterations revealed by EEG and/or brain imaging analysis are studied in ASD”? Additionally, secondary research questions arising from the review were explored: (i) what are the altered brain regions associated with genetic alterations in ASD? and (ii) which methods are more frequently used to study brain alterations associated with genetics in ASD?

### 2.2. Identification of Relevant Studies

Based on the aim of this review, an academic search was conducted in Pubmed (https://pubmed.ncbi.nlm.nih.gov) and EBSCOHOST (https://www.ebsco.com) on 12 April 2023, using the search syntax terms: “Autism OR ASD OR Asperger”; AND “EEG OR fMRI OR MRI OR MRS OR electroencephalography OR functional magnetic resonance imaging OR magnetic resonance imaging OR magnetic resonance spectroscopy” AND “Genomics OR genetic variant OR genetic variation OR Genetics OR Mutation OR CNV OR Copy Number Variant OR SNV OR Single Nucleotide Variant”. Search terms were combined using Boolean logic, as referred to in the syntax above. Additionally, a filter for the “year” was applied to each database search, including only those articles published from 2000 to 2022, a period during which there was a noticeable increase in neuroimaging research, particularly in the domain of psychopathology [30]. This filtering was also due to the limitations of MRI resolution prior to 2000 [31]. Furthermore, an additional filter was applied, limiting the results to peer-reviewed and published scientific papers and focusing on English language publications involving human subjects.

### 2.3. Study Selection

At first, titles and abstracts for each article were reviewed to determine whether the studies included the terms pre-defined. After selecting the first set of studies, a full-text reading of the remaining articles was performed.

Studies were included if they met the following inclusion criteria:

(1) Empirical research published in peer-reviewed journals;
(2) Included patients with ASD diagnosis (when the study refers to diagnostic instruments, or uses a clinical cohort or known database of subjects with ASD), and an identified genetic alteration;
(3) Described brain measures assessed by EEG, fMRI, MRI or MRS;
(4) Were published in English.

Studies were excluded as determined by the following criteria:

(1) The reported data were obtained by methods that were not MRI or EEG;
(2) The sample included individuals without an ASD diagnosis (e.g., with autistic-like traits, children with ASD risk);
(3) Case-report studies;
(4) Review studies;
(5) Studies in animal models.

### 2.4. Charting the Data: Structure of the Findings

For each study, dimensions that potentially impacted results and conclusions were extracted by two of the authors, such as sample characteristics (e.g., age, diagnostic confirmation method, sample size), method (e.g., EEG, fMRI) and brain area outcomes. This extraction of data should be able to describe the research context and possible heterogeneities of the data, expressed in a standardized way to allow further comparison and interpretation.

## 3. Results and Discussion

**Figure 1:**
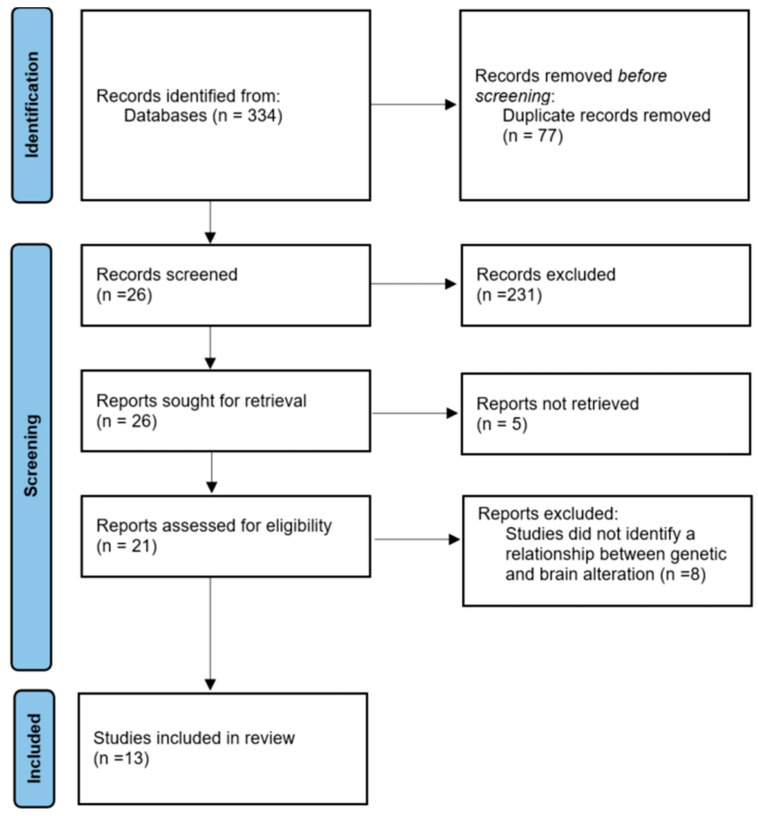
PRISMA (Preferred Reporting Items for Systematic Reviews and Meta-Analyses) flowchart describing the papers selection process.

After performing title and abstract screening (n = 257), and full-text review (n = 26), there was a final selection of 13 eligible papers with a good strength of agreement (α = 0.885). The complete screening process is presented in Figure 1.

**Figure 2:**
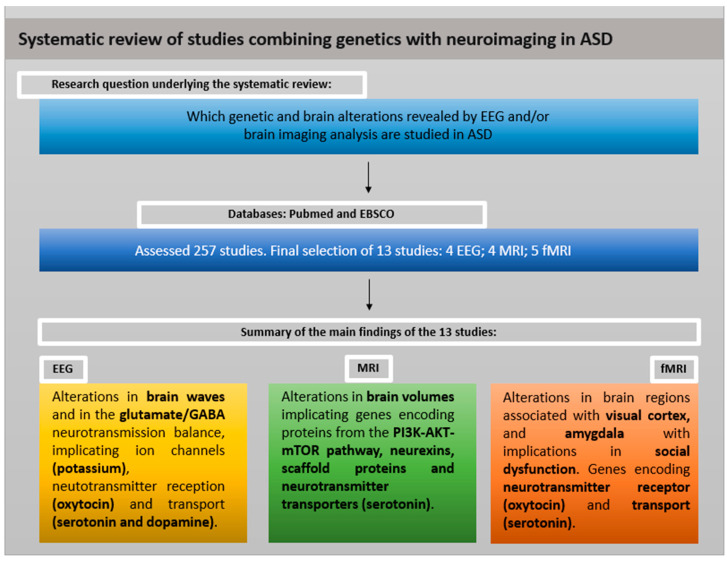
Overview of the systematic review. EEG: electroencephalography; MRI: magnetic resonance imaging; fMRI: functional magnetic resonance imaging; GABA: gamma-aminobutyric acid.

A large percentage of the 13 selected studies were conducted in the USA (38%), with the remaining studies distributed among China (23%), Europe (15%), Canada (8%), Israel (8%) and Iran (8%) (see Supplementary Table S1 for a list of studies). The year of publication ranged from 2007 to 2022. Sample sizes varied greatly, involving between 2 and 916 participants [32,33], with 77% of the studies having an average of 34 participants. Globally, the studies included a total of 1819 participants, including children, adolescents and adults with ASD (range, 2–64 years). Among the 13 studies, 9 are case–control studies, 1 is a family study, 1 is a longitudinal study and 2 are focused on a group of cases. An overview of the systematic review is shown in Figure 2 and a summary of the results using brain analysis method is shown in Table 1.

All 13 studies identified either a brain structural or functional alteration related with the presence of genetic variation or with alterations in gene expression (see Supplementary Table S1 for the complete results). Eight studies analyze data on genetic variation (mutations, polymorphisms or other variants) and five studies analyze alterations in gene expression. The EEG studies describe brain alterations and variants in the genes *FOXP2*, *KCNJ10*, *SLC6A4*, *SLC6A3*, *NLGN4X*, *GLRB* and *ANK3* (Table 1). Haghighatfard et al. [34] found a significant correlation between the decreased expression of *FOXP2* and low alpha and gamma bands (waves between the frequencies 7.5 and 9.25 Hz and between the frequencies 30 and 200 Hz, respectively) in the frontal lobe, and between the decreased expression of *FOXP2* and high theta bands (waves between the frequencies 3.5 and 6 Hz) in the occipital lobe of children with ASD. Alterations in slow waves (between the frequencies 0.5 and 4 Hz) in EEG, which are indicative of cerebral dysfunction, were identified by Cucchiara et al. [35] in subjects carrying variants in the gene *KCNJ10*, which encodes a member of the potassium channel Kir4.1. Sjaarda et al. [36] reported an association of the 5-HTTLPR functional polymorphism located in the promoter of the *SLC6A4* gene, with increased latency in the time taken by the stimulus information to generate the P1 component of the EEG response, which measures sensitivity to faces. Additionally, the authors found that *SLC6A3* polymorphisms correlate with the reduced amplitude of the N170 EEG component, another marker of face-sensitivity processing, in male participants with ASD. Furthermore, Bonnet-Brilhault et al. [32] detected an atypical electrophysiological pattern targeting glutamate/GABA neurotransmission in ASD individuals carrying *NLGN4X* gene variants with additional variants in *GLRB* or *ANK3* genes.

Three MRI studies detected alterations in brain volumes related with *CNTNAP2*, *PPP2RD*, *PTEN* and *SLC6A4* genes, and in brain morphology related with the *SHANK3* gene (Table 1). Chien et al. [37] reported that individuals with ASD showed smaller cortical thickness in bilateral cingulate subregions when compared to controls and identified polymorphisms in the *CNTNAP2* gene significantly associated with the white matter volume of the right caudal anterior cingulate gyrus. Li et al. [38] reported that ASD children with *SHANK3* deletions and/or mutations showed more alterations in the regions of the dorsolateral prefrontal cortex, inferior frontal cortex auditory areas, lateral temporal cortex, inferior parietal gyrus, dorsal visual streams, temporo-parieto-occipital junction, parahippocampus, orbitofrontal cortex, anterior cingulate cortex, medial visual areas and parieto-occipital sulcus, when compared with ASD children without these genetic alterations. Yeung et al. [39] studied three children with both macrocephaly and ASD, carrying variants in three genes from the PI3K-AKT-mTOR pathway: one child with a variant in the *PPP2R5D* gene, a second child, who also has megalencephaly, with a variant in the *PIK3CA* gene, and a third child with a variant in the *PTEN* gene. Wassink et al. [40] identified a *SLC6A4* genotype in the 5-HTTLPR promoter region, which influences cerebral cortical gray matter volumes in individuals with ASD.

Five fMRI studies reported alterations in brain connectivity associated with *FILIP1*, *GABRQ* and *SLC6A4* gene expression, and *OXTR* polymorphisms (Table 1). Berto et al. [33] identified a subset of upregulated genes during the adult stages of life that showed delayed upregulation in individuals with ASD when compared with controls. This subset of genes is enriched in voltage-gated ion channels and inhibitory neurons, suggesting excitatory-inhibitory imbalances in ASD. The authors further assessed differences in gene expression in brain regions and showed that the primary visual cortex is the most affected region by an excitation–inhibition imbalance in individuals with ASD. The genes with the highest effect size on functional connectivity detected using fMRI are the *FILIP1* and *GABRQ* genes. Uzefovsky et al. [41] found two *OXTR* genotypes associated with significant hyperactivation in the right supramarginal gyrus (rSMG) and the right inferior parietal lobule (rIPL). In emotional recognition tasks, Velasquez et al. [42] reported that individuals with ASD with low expressing 5-HTTLPR genotypes showed significantly greater connectivity than individuals with ASD with higher expressing genotypes, and TD individuals. Moreover, the authors showed that individuals with ASD carrying higher expressing genotypes exhibit a negative relationship between amygdala–subgenual anterior cingulate cortex (sACC) connectivity and social dysfunction. Wiggins et al. [43] identified the occurrence of stronger connectivity in low versus high expressing *SLC6A4* gene promoter region (5-HTTLPR) genotypes in individuals with ASD. The authors also reported 5-HTTLPR genotypes that differ between cases and controls, related with the amygdala habituation to sad faces. Amygdala habituation is a mechanism of fast decrease in amygdala responsiveness to the repeated presentation of stimuli, which is key for maintaining adaptive levels of response to predictable social stimuli [45]. 

The brain regions analyzed by the selected publications to evaluate the performance in these mechanisms were the visual cortex and the occipital lobe, and subcortical areas such as the amygdala. Difficulties in face identification and emotion recognition have implications in social interactions and contribute substantially to the diminished attention to human faces observed in ASD patients [46,47].

EEG has been used to examine the functional connectivity across different brain regions in ASD, as well as the integrity of structural connections between brain areas [24,26,27] or alterations in glutamate/GABA neurotransmission and brain mechanisms involved in facial emotion recognition [48]. The gene variants found in individuals with ASD in Bonnet-Brilhault et al. [32] are related with an atypical electrophysiological pattern involving glutamate/GABA neurotransmission and involves the gene *NLGN4X*, which encodes a neuroligin (neuronal cell surface protein). Evidence shows that this gene is implicated in the formation and remodeling of central nervous system synapses [49,50].

Alterations in measures of brain size and volumes are some of the brain features most frequently evaluated in the studies selected [37,39,40]. Macrocephaly, which is defined as a head circumference greater than or equal to more than two standard deviations, was one of the features analyzed as it is commonly observed in individuals with ASD. The study addressing this question in the present review [39] shows that three children with macrocephaly and ASD have variants in the genes *PPP2R5D*, *PIK3CA* or *PTEN*. These genes belong to the PI3K/AKT/mTOR pathway, an intracellular signaling pathway that is key for cell cycle regulation. Variants in the *PTEN* gene are a well-known hallmark of many ASD patients with macrocephaly; however, the role of other genes in macrocephaly has also been investigated. The *PPP2R5D* gene is implicated in the negative control of cell growth and division, and *PIK3CA* is an oncogene that was shown to be implicated in cervical cancers [51]. These observations suggest that the dysregulation of mechanisms involved in cell division and proliferation may underlie brain overgrowth in individuals with ASD with macrocephaly. Another synaptic gene important for brain development and function, *CNTNAP2*, was also associated with thinner cortical thickness in bilateral cingulate subregions [37]. Alterations of the cingulate structure are frequently reported in ASD, and evidence suggests that alterations in the activity of the anterior cingulate cortex involves social cognition dysfunction [52], one of the core characteristics of ASD. The synaptic gene *SHANK3* was also found to be related with cortical brain alterations [38]. Children with ASD with *SHANK3* mutations showed more abnormalities in cortical regions [38], which are involved in memory and spatial processing.

Most of the genes reported in the selected publications encode proteins that intervene in synaptic and neurotransmitter mechanisms, such as the genes *OXTR*, *SLC6A3* and *SLC6A4*, or encode scaffold proteins such as the *SHANK3* gene, which interacts with several proteins and complexes to coordinate dendritic spine and synapse formation, maturation and maintenance. OXTR is a G-protein coupled receptor for oxytocin, a neurotransmitter with an important role in the regulation of social behaviors and social stress [53]. Several polymorphisms within the *OXTR* gene have previously been associated with ASD risk [54,55,56,57], highlighting the importance of *OXTR* for the disease. The *SLC6A3* gene encodes the dopamine transporter. There is ample evidence that the dopaminergic system has a key role in reward processing and learning [58,59] and that deficits in the dopamine reward system lead to social motivation impairments in individuals with ASD [60], while polymorphisms in *SLC6A3* may be a risk factor for ASD [61]. Many previous studies also implicated the *SLC6A4* gene, which encodes the serotonin transporter, with emotions, social cognition, and behavior. For instance, genetic variants in the *SLC6A4* promoter 5-HTTLPR region are associated with reduced serotonin transporter activity and increased levels of anxiety [62], and with areas of the brain involved in social cognition [63]. Specific variants in this gene are also associated with hyperserotonemia, which has consistently been described in about one third of ASD subjects [64]. These observations suggest that dysfunction in the serotonergic system may cause social and communication deficits in ASD [65]. Overall, *SLC6A4* is the gene more frequently implicated in functional or morphological alterations observed in the selected studies, and the results highlight a role in amygdala connectivity [42,43,44] and an association with increased cortical gray matter volumes [40].

Some of the studies included in this review need to be replicated with a larger number of participants to verify the consistency of the results. It is crucial to design more studies with larger sample sizes as the interpretation of neurogenetic studies has been often hindered by relatively small sample sizes and a lack of replication across different studies. An additional limitation of this review is the absence of information concerning the sex of participants in the majority of the included studies. The previous literature has highlighted sex differences in childhood social cognition and brain development, especially at specific developmental stages [66], but this aspect is not contemplated in the majority of the studies. Despite these limitations, the results of this systematic review suggest that integrating results from genetic, neuroimaging and clinical phenotypes has promising potential to better understand the etiology and physiopathology of ASD.

## 4. Conclusions

The impact of specific genetic variants and alterations of gene expression patterns from neurotransmission and synaptic genes in brain structure and function is highlighted in this review. A set of studies dedicated to understanding the links between genetic alterations and brain morphology and function was identified. The techniques reviewed, namely EEG, MRI and fMRI, provide windows to understand brain development and function; however, these types of studies still have many feasibility limitations, because they are costly and logistically complex. The resulting small sample sizes, compounded by the complex genetic architecture of ASD, hinder the consistency of findings across studies and the capacity to establish firm conclusions. In view of these limitations, it is therefore remarkable that some results tend to be consistent across reports, and reinforce previous evidence gathered from very different types of studies. This is the case for instance for the dopamine and serotonin transporter genes, or the genes involved in the PI3K/AKT/mTOR pathway, or the *OXTR* gene. A role for variation in neurexins and neuroligins genes, which have also been solidly implicated in ASD genetic risk, is also reinforced by this review. Viewed from this perspective, it is likely that brain imaging and EEG can provide precious support to understand the biological consequences of genetic variants identified from high throughput genomic methods. For any complex multifactorial disorder, including ASD, identifying relevant genetic variants is no longer a problem with high throughput sequencing, enormous sample sizes and inference from ever more sophisticated statistical analysis methods. However, functional analysis is still necessary to proof the functional consequence from any genetic variant. The promising findings from this review provide support for increasing the employment of brain imaging and EEG methodology to understand the functional consequences of genetic variants in the brain.

Overall, this review shows that genetic risk variants and altered patterns of gene expression defined in individuals with ASD affect brain function and structure. The brain circuits implicated are associated with face processing and social behavior, excitation–inhibition balance and anomalies in brain volumes. The clarification of the impact of genetic alterations in ASD in relation to brain phenotypes will be key in defining effective treatments that target particular genetic profiles related with the brain regions and functions affected. The integration of genetic studies and neuroimaging studies in ASD can therefore greatly contribute to elucidating the brain pathways underlying the phenotypic and clinical heterogeneity that characterizes this disorder.

## Figures and Tables

**Table 1:** Summary of the main findings of the systematic review using brain analysis method. The term “mutation” is used to refer to gene variants that have a known damaging or pathogenic effect in proteins, while gene variants that occur in a population with a frequency of 1% or higher are designated as “polymorphisms”.

Summary of Findings of EEG Studies					
Sample Size (Cases)	Brain Area Analyzed	Gene/Genomic Region	Main Results	Genetic Analysis	Study
n = 2	Whole brain analysis	*NLGN4X*, *GLRB* and *ANK3*	Gene variants implicated in atypical electrophysiological pattern targeting glutamate/GABA neurotransmission.	Gene variants	Bonnet-Brilhault et al., 2016 [32]
n = 450	Frontal and occipital lobe	*FOXP2*	Significant correlation between decreased *FOXP2* expression and alpha, gamma and theta bands.	Gene expression	Haghighatfard et al., 2022 [34]
n = 14	Fronto-central bipolar EEG derivations	*KCNJ10*	Period-amplitude slow wave features are modified in subjects carrying variants in the *KCNJ10* gene.	Gene variants	Cucchiara et al., 2020 [35]
n = 50	Mean amplitude and latency of the P1 and N170 components	*COMT*, *OXTR*, *SLC6A4* and *SLC6A3*	*SLC6A4* polymorphisms were associated with increased P1 latency. *SLC6A3* polymorphisms associated with reduced N170 amplitude.	Polymorphisms	Sjaarda et al., 2019 [36]
**Summary of findings of MRI studies**					
Sample size (cases)	Brain area analyzed	Gene/genomic region	Main results	Genetic Analysis	Study
n = 118	Cortex	*CNTNAP2*	Thinner cortical thickness in bilateral cingulate subregions. Polymorphisms associated with the white matter volume of the right caudal anterior cingulate gyrus.	Polymorphisms	Chien et al., 2021 [37]
n = 36	Cortex	*SHANK3*	ASD individuals with *SHANK3* mutations have significant increase in cortical thickness.	Mutations	Li et al., 2021 [38]
n = 10	Megalencephaly, polymicrogyria and periventricular white matter signal abnormalities. Ventriculomegaly.	*PIK3CA*, *PTEN*, *MTOR* and *PPP2R5D*	Macrocephaly and megalencephaly related with variants in genes from the PI3K-AKT-mTOR pathway.	Gene variants	Yeung et al., 2017 [39]
n = 44	Cerebral cortical and cerebellar gray and white matter volumes	*SLC6A4*	*SLC6A4* genotype is associated with cerebral cortical gray matter volumes.	Gene variants	Wassink et al., 2007 [40]
**Summary of findings of fMRI studies**					
Sample size (cases)	Brain area analyzed	Gene/genomic region	Main results	Genetic Analysis	Study
n = 916	Several	Brain transcriptome	Genes enriched in voltage-gated ion channels and inhibitory neurons are related with excitation–inhibition imbalance in ASD. The primary visual cortex is the most affected region. Genes with	Gene expression	Berto et al., 2022 [33]
			highest effect size: *FILIP1* and *GABRQ.*		
n = 38	Whole brain analysis	*OXTR*	Genotypes associated with the right supramarginal gyrus (rSMG) and the right inferior parietal lobule (rIPL).	Polymorphisms	Uzefovsky et al., 2019 [41]
n = 43	Amygdala	*SLC6A4*	Expression levels of different genotypes related with the amygdala and subgenual anterior cingulate cortex (amygdala-sACC) connectivity and with social dysfunction.	Gene expression	Velasquez et al., 2017 [42]
n = 54	Posterior-anterior default network	*SLC6A4*	Stronger connectivity in low versus high expressing genotypes in ASD.	Gene expression	Wiggins et al., 2013 [43]
n = 44	Amygdala	*SLC6A4*	Genotypes related with amygdala habituation to sad faces differs in the ASD group vs. controls.	Gene expression	Wiggins et al., 2014 [44]

## Data Availability

Data sharing not applicable.

## Supplementary Materials

The supporting information can be downloaded at: https://www.mdpi.com/article/10.3390/ijms25094938/s1.

## References

[1] American Psychiatric Association (2013). Diagnostic and Statistical Manual of Mental Disorders: DSM-5.

[2] Rosenberg R.E., Law J.K., Yenokyan G., McGready J., Kaufmann W.E., Law P.A. (2009). Characteristics and concordance of autism spectrum disorders among 277 twin pairs. Arch. Pediatr. Adolesc. Med..

[3] Ozonoff S., Young G., Carter A., Messinger D., Yirmiya N., Zwaigenbaum L., Bryson S., Carver L., Constantino J., Dobkins K. (2011). Recurrence Risk for Autism Spectrum Disorders: A Baby Siblings Research Consortium Study. Pediatrics.

[4] Tick B., Bolton P., Happé F., Rutter M., Rijsdijk F. (2016). Heritability of autism spectrum disorders: A meta-analysis of twin studies. J. Child Psychol. Psychiatry.

[5] Bai D., Yip B., Windham G., Sourander A., Francis R., Yoffe R., Glasson E., Mahjani B., Suominen A., Leonard H. (2019). Association of Genetic and Environmental Factors with Autism in a 5-Country Cohort. JAMA Psychiatry.

[6] O’Roak B.J., Vives L., Girirajan S., Karakoc E., Krumm, Coe B., Levy R., Ko A., Lee C., Smith J. (2012). Sporadic autism exomes reveal a highly interconnected protein network of de novo mutations. Nature.

[7] O’Roak B.J., Stessman H., Boyle E., Witherspoon K., Martin B., Lee C., Vives L., Baker C., Hiatt J., Nickerson D. (2014). Recurrent de novo mutations implicate novel genes underlying simplex autism risk. Nat. Commun..

[8] Ramaswami G., Geschwind D.H. (2018). Genetics of autism spectrum disorder. Handb. Clin. Neurol..

[9] Satterstrom F.K., Kosmicki J., Wang J., Breen M., De Rubeis S., An J., Peng M., Collins R., Grove J., Klei L. (2020). Large-Scale Exome Sequencing Study Implicates Both Developmental and Functional Changes in the Neurobiology of Autism. Cell.

[10] Cirnigliaro M., Chang T.S., Arteaga S.A., Pérez-Cano L., Ruzzo E.K., Gordon A., Bicks L.K., Jung J.Y., Lowe J.K., Wall D.P. (2023). The contributions of rare inherited and polygenic risk to ASD in multiplex families. Proc. Natl. Acad. Sci. USA.

[11] Montanari M., Martella G., Bonsi P., Meringolo M. (2022). Autism Spectrum Disorder: Focus on Glutamatergic Neurotransmission. Int. J. Mol. Sci..

[12] De Rubeis S., He X., Goldberg A., Poultney C., Samocha K., Cicek A., Kou Y., Liu L., Fromer M., walker S. (2014). Synaptic, transcriptional and chromatin genes disrupted in autism. Nature.

[13] Geschwind D.H., State M.W. (2015). Gene hunting in autism spectrum disorder: On the path to precision medicine. Lancet Neurol..

[14] Hashem S., Nisar S., Bhat A., Yadav S., Azeem M., Bagga P., Fakhro K., Reddy R., Frenneaux M., Haris M. (2020). Genetics of structural and functional brain changes in autism spectrum disorder. Transl. Psychiatry.

[15] Xie Y., Zhang X., Liu F., Qin W., Fu J., Xue K., Yu C. (2020). Brain mRNA Expression Associated with Cortical Volume Alterations in Autism Spectrum Disorder. Cell Rep..

[16] Mahajan R., Mostofsky S.H. (2015). Neuroimaging endophenotypes in autism spectrum disorder. CNS Spectr..

[17] Ali M.T., ElNakieb Y., ElNakieb A., Shalaby A., Mahmoud A., Ghazal M., Yousaf J., Khalifeh H., Casanova M., Barnes G. (2022). The Role of Structure MRI in Diagnosing Autism. Diagnostics.

[18] Amaral D.G., Schumann C.M., Nordahl C.W. (2008). Neuroanatomy of autism. Trends Neurosci..

[19] Panizzon M.S., Fennema-Notestine C., Eyler L., Jernigan T., Prom-Wormley E., Neale M., Jacobson K., Lyons M., Grant M., Franz C. (2009). Distinct genetic influences on cortical surface area and cortical thickness. Cereb. Cortex.

[20] Webb S.J., Sparks B., Friedman S., Shaw D., Giedd J., Dawson G., Dager S. (2009). Cerebellar vermal volumes and behavioral correlates in children with autism spectrum disorder. Psychiatry Res. Neuroimaging.

[21] Geschwind D.H., Levitt P. (2007). Autism spectrum disorders: Developmental disconnection syndromes. Curr. Opin. Neurobiol..

[22] Cantor D.S., Thatcher R.W., Hrybyk M., Kaye H. (1986). Computerized EEG analyses of autistic children. J. Autism Dev. Disord..

[23] Minshew N.J., Williams D.L. (2007). The new neurobiology of autism: Cortex, connectivity, and neuronal organization. Arch. Neurol..

[24] Wass S. (2011). Distortions and disconnections: Disrupted brain connectivity in autism. Brain Cogn..

[25] Mak-Fan K.M., Morris D., Vidal J., Anagnostou E., Roberts W., Taylor M.J. (2013). White matter and development in children with an autism spectrum disorder. Autism.

[26] Olejniczak P. (2006). Neurophysiologic basis of EEG. J. Clin. Neurophysiol..

[27] Milovanovic M., Grujicic R. (2021). Electroencephalography in Assessment of Autism Spectrum Disorders: A Review. Front. Psychiatry.

[28] Page M.J., McKenzie J.E., Bossuyt P.M., Boutron I., Hoffmann T.C., Mulrow C.D., Shamseer L., Tetzlaff J.M., Akl E.A., Brennan S.E. (2021). The PRISMA 2020 statement: An updated guideline for reporting systematic reviews. BMJ.

[29] Moher D., Liberati A., Tetzlaff J., Altman D.G. (2020). Preferred reporting items for systematic reviews and meta-analyses: The PRISMA statement. Int. J. Surg..

[30] McLoughlin G., Palmer J.A., Rijsdijk F., Makeig S. (2014). Genetic Overlap between Evoked Frontocentral Theta-Band Phase Variability, Reaction Time Variability, and Attention-Deficit/Hyperactivity Disorder Symptoms in a Twin Study. Biol. Psychiatry.

[31] FChance S., Abbott L.F., Reyes A.D. (2002). Gain Modulation from Background Synaptic Input. Neuron.

[32] Bonnet-Brilhault F., Alirol S., Blanc R., Bazaud S., Marouillat S., Thépault R., Andres C., Lemmonier É., Barthélémy C., Raynaud M. (2016). GABA/Glutamate synaptic pathways targeted by integrative genomic and electrophysiological explorations distinguish autism from intellectual disability. Mol. Psychiatry.

[33] Berto S., Treacher A., Caglayan E., Luo D., Haney J., Gandal M., Geschwind D., Montillo A., Konopka G. (2022). Association between resting-state functional brain connectivity and gene expression is altered in autism spectrum disorder. Nat. Commun..

[34] Haghighatfard A., Asl E., Bahadori R., Aliabadian R., farhadi M., Mohammadpour F., Tabrizi Z. (2022). FOXP2 down expression is associated with executive dysfunctions and electrophysiological abnormalities of brain in Autism spectrum disorder; a neuroimaging genetic study. Autism. Dev. Lang Impair..

[35] Cucchiara F., Frumento P., Banfi T., Sesso G., Galante M., D’Ascanio P., Valvo G., Sicca F., Faraguna U. (2020). Electrophysiological features of sleep in children with Kir4.1 channel mutations and Autism-Epilepsy phenotype: A preliminary study. Sleep.

[36] Sjaarda C.P., Sabbagh M., Wood D., Ward-King J., McNaughton A., Hudson M., Tao M., Ayub M., Liu X. (2019). Homozygosity for the 10-repeat dopamine transporter (DAT1) allele is associated with reduced EEG response in males with ASD. Res. Autism Spectr. Disord..

[37] Chien Y.-L., Chen Y.-C., Gau S.S.-F. (2021). Altered cingulate structures and the associations with social awareness deficits and CNTNAP2 gene in autism spectrum disorder. NeuroImage Clin..

[38] Li D., Liu C., Huang Z., Li H., Xu Q., Zhou B., Hu C., Zhang Y., Wang Y., Nie J. (2021). Common and Distinct Disruptions of Cortical Surface Morphology between Autism Spectrum Disorder Children with and without SHANK3 Deficiency. Front. Neurosci..

[39] Yeung K.S., Tso W., Ip J., Mak C., Leung G., Tsang M., Ying D., Pei S., Lee S., Yang W. (2017). Identification of mutations in the PI3K-AKT-mTOR signalling pathway in patients with macrocephaly and developmental delay and/or autism. Mol. Autism.

[40] Wassink T.H., Hazlett H.C., Epping E.A., Arndt S., Dager S.R., Schellenberg G.D., Dawson G., Piven J. (2007). Cerebral cortical gray matter overgrowth and functional variation of the serotonin transporter gene in autism. Arch. Gen. Psychiatry.

[41] Uzefovsky F., Bethlehem R.A.I., Shamay-Tsoory S., Ruigrok A., Holt R., Spencer M., Chura L., Warrier V., Chakrabarti B., Bullmore E. (2019). The oxytocin receptor gene predicts brain activity during an emotion recognition task in autism. Mol. Autism.

[42] Velasquez F., Wiggins J.L., Mattson W.I., Martin D.M., Lord C., Monk C.S. (2017). The influence of 5-HTTLPR transporter genotype on amygdala-subgenual anterior cingulate cortex connectivity in autism spectrum disorder. Dev. Cogn. Neurosci..

[43] Wiggins J.L., Peltier S.J., Bedoyan J.K., Carrasco M., Welsh R.C., Martin D.M., Lord C., Monk C.S. (2013). The impact of serotonin transporter genotype on default network connectivity in children and adolescents with autism spectrum disorders. NeuroImage Clin..

[44] Wiggins J.L., Swartz J.R., Martin D.M., Lord C., Monk C.S. (2014). Serotonin transporter genotype impacts amygdala habituation in youth with autism spectrum disorders. Soc. Cogn. Affect. Neurosci..

[45] Swartz J.R., Wiggins J.L., Carrasco M., Lord C., Monk C.S. (2013). Amygdala Habituation and Prefrontal Functional Connectivity in Youth with Autism Spectrum Disorders. J. Am. Acad. Child Adolesc. Psychiatry.

[46] Hobson J.A., Stickgold R., Pace-Schott E.F. (1998). The neuropsychology of REM sleep dreaming. NeuroReport.

[47] Bird G., Catmur C., Silani G., Frith C., Frith U. (2006). Attention does not modulate neural responses to social stimuli in autism spectrum disorders. NeuroImage.

[48] Black M.H., Chen N.T.M., Iyer K.K., Lipp O.V., Bölte S., Falkmer M., Tan T., Girdler S. (2017). Mechanisms of facial emotion recognition in autism spectrum disorders: Insights from eye tracking and electroencephalography. Neurosci. Biobehav. Rev..

[49] Yumoto T., Kimura M., Nagatomo R., Sato T., Utsunomiya S., Aoki N., Kitaura M., Takahashi K., Takemoto H., Watanabe H. (2020). Autism-associated variants of neuroligin 4X impair synaptogenic activity by various molecular mechanisms. Mol. Autism.

[50] Cast T.P., Boesch D.J., Smyth K., Shaw A.E., Ghebrial M., Chanda S. (2021). An Autism-Associated Mutation Impairs Neuroligin-4 Glycosylation and Enhances Excitatory Synaptic Transmission in Human Neurons. J. Neurosci..

[51] Voutsadakis I.A. (2021). PI3KCA Mutations in Uterine Cervix Carcinoma. J. Clin. Med..

[52] Cauda F., D’Agata F., Sacco K., Duca S., Geminiani G., Vercelli A. (2011). Functional connectivity of the insula in the resting brain. NeuroImage.

[53] Heinrichs M., Domes G., Neumann I.D., Landgraf R. (2008). Neuropeptides and social behaviour: Effects of oxytocin and vasopressin in humans. Progress in Brain Research.

[54] Wu S., Jia M., Ruan Y., Liu J., Guo Y., Shuang M., Gong X., Zhang Y., Yang X., Zhang D. (2005). Positive association of the oxytocin receptor gene (OXTR) with autism in the Chinese Han population. Biol. Psychiatry.

[55] Campbell D.B., Datta D., Jones S.T., Batey Lee E., Sutcliffe J.S., Hammock E.A., Levitt P. (2011). Association of oxytocin receptor (OXTR) gene variants with multiple phenotype domains of autism spectrum disorder. J. Neurodevelop. Disord..

[56] Liu X., Kawashima M., Miyagawa T., Otowa T., Latt K.Z., Thiri M., Nishida H., Sugiyama T., Tsurusaki Y., Matsumoto N. (2015). Novel rare variations of the oxytocin receptor (OXTR) gene in autism spectrum disorder individuals. Hum. Genome Var..

[57] Al-Ali Z., Yasseen A.A., Al-Dujailli A., Al-Karaqully A.J., McAllister K.A., Jumaah A.S. (2022). The oxytocin receptor gene polymorphism rs2268491 and serum oxytocin alterations are indicative of autism spectrum disorder: A case-control paediatric study in Iraq with personalized medicine implications. PLoS ONE.

[58] Alcaro A., Huber R., Panksepp J. (2007). Behavioral functions of the mesolimbic dopaminergic system: An affective neuroethological perspective. Brain Res. Rev..

[59] Lewis R.G., Florio E., Punzo D., Borrelli E., Engmann O., Brancaccio M. (2021). The Brain’s Reward System in Health and Disease. Circadian Clock in Brain Health and Disease.

[60] Dawson G., Webb S.J., Wijsman E., Schellenberg G., Estes A., Munson J., Faja S. (2005). Neurocognitive and electrophysiological evidence of altered face processing in parents of children with autism: Implications for a model of abnormal development of social brain circuitry in autism. Dev. Psychopathol..

[61] Azzam A.A.A., Bahgat D.M.R., Shahin R.M.H., Nasralla R.M.A. (2018). Association study between polymorphisms of dopamine transporter gene (SLC6A3), dopamine D1 receptor gene (DRD1), and autism. J. Med. Sci. Res..

[62] Lesch K.P., Bengel D., Heils A., Sabol S.Z., Greenberg B.D., Petri S., Benjamin J., Müller C.R., Hamer D.H., Murphy D.L. (1996). Association of anxiety-related traits with a polymorphism in the serotonin transporter gene regulatory region. Science.

[63] Canli T., Omura K., Haas B.W., Fallgatter A., Constable R.T., Lesch K.P. (2005). Beyond affect: A role for genetic variation of the serotonin transporter in neural activation during a cognitive attention task. Proc. Natl. Acad. Sci. USA.

[64] Coutinho A.M., Oliveira G., Morgadinho T., Fesel C., Macedo T.R., Bento C., Marques C., Ataíde A., Miguel T., Borges L. (2004). Variants of the serotonin transporter gene (SLC6A4) significantly contribute to hyperserotonemia in autism. Mol. Psychiatry.

[65] Canli T., Lesch K.-P. (2007). Long story short: The serotonin transporter in emotion regulation and social cognition. Nat. Neurosci..

[66] Etchell A., Adhikari A., Weinberg L.S., Choo A.L., Garnett E.O., Chow H.M., Chang S.E. (2018). A systematic literature review of sex differences in childhood language and brain development. Neuropsychologia.

